# Supplementary material for: Identification of photoperiod-induced specific miRNAs in the adrenal glands of Sunite sheep (Ovis aries)
Source: Front Vet Sci. 2022 Jul 22;9:888207. doi: 10.3389/fvets.2022.888207 (PMC9354845; doi:10.3389/fvets.2022.888207)
Supplement: Supplementary file 1 [file Data_Sheet_1.ZIP › Supplementary file/miRDeep2 file.zip!/miRDeep2 file/Novel_Structure/SPLP42A3/Novel_1.pdf]

Provisional ID	:	NC_019468.2_20889
Score total	:	5.8
Score for star read(s)	:	3.9
Score for read counts	:	-1.3
Score for mfe	:	1.7
Score for randfold	:	1.6
Score for cons. seed	:	
Total read count	:	9
Mature read count	:	6
Loop read count	:	0
Star read count	:	3

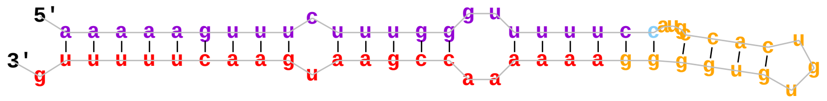

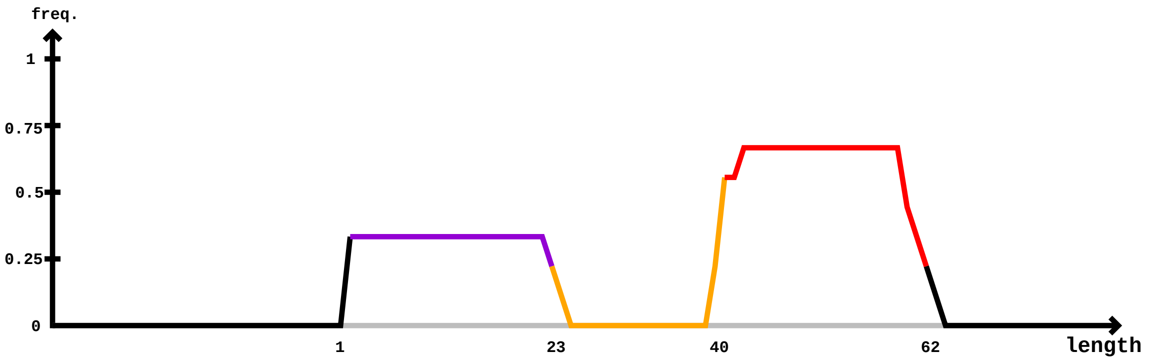


```
                                 Star                                  Mature
5'- caguggaaguaauuuguauuggguugaccaaaaaguuucuuuggguuuuuccaugccacuguguggggaaaaaaccgaaugaacuuuuuggccaacacaauauuuucuu   -3'   obs
    caguggaaguaauuuguauuggguugaccaaaaaguuucuuuggguuuuuccaugccacuguguggggaaaaaaccgaaugaacuuuuuggccaacacaauauuuucuu         exp
    ....(((((......((((((.((((.(((((((((((.(((((..((((((...((((...))))))))))..))))).))))))))))).)))).))))))))))).   reads   mm   sample
    .............................aaaaaguuuGuuuggguuuuu..........................................................   1       1    seq
    .............................aaaaaguuuGuuuggguuuuuc.........................................................   2       1    seq
    .................................................................gaaaaaCccgaaugaacuuu.......................   1       1    seq
    .................................................................gaaaaaCccgaaugaacuuuu......................   1       1    seq
    ..................................................................aaaaaCccgaaugaacuuu.......................   1       1    seq
    ..................................................................aaaaaCccgaaugaacuuuuug....................   2       1    seq
    ....................................................................aaaaccgaaugaacuuuuu.....................   1       0    seq
```
